# Relationship between Emotional Intelligence, Cybervictimization, and Academic Performance in Secondary School Students

**DOI:** 10.3390/ijerph17217717

**Published:** 2020-10-22

**Authors:** Ana María Martínez-Martínez, Remedios López-Liria, José Manuel Aguilar-Parra, Rubén Trigueros, María José Morales-Gázquez, Patricia Rocamora-Pérez

**Affiliations:** 1 Department of Education, University of Almería, 04120 Almería, Spain; 2 Hum-498 Research Team, Health Research Centre, Department of Nursing Science, Physiotherapy and Medicine, University of Almería, 04120 Almería, Spain; rocamora@ual.es; 3 Hum-878 Research Team, Health Research Centre, Department of Psychology, University of Almería, 04120 Almería, Spain; rtr088@ual.es; 4 Department of Nursing, University of Las Palmas de Gran Canaria (ULPGC), Juan de Quesada, 30, 35001 Las Palmas de Gran Canaria, Spain; mariajose.morales@ulpgc.es

**Keywords:** cybervictimization, emotional intelligence, academic performance

## Abstract

The benefits attributed to emotional intelligence (EI) in a school environment can be observed in areas such as interpersonal relationships, psychological well-being, academic performance, and avoidance of disruptive behaviors. The objective of this study was to analyze a sample of 3451 adolescents from a secondary school to test whether EI is a protector against cybervictimization and the repercussions of cybervictimization, and whether EI has an influence on academic performance. The instruments used in the study included a questionnaire of risk factors for cybervictimization—the Trait Meta Mood Scale 24 (Spanish version)—and the global marks or academic performance of the students. The relationships between the variables were analyzed and a structural equation model was developed. The correlations revealed that there was a positive relationship between EI and student academic performance, but there was also a negative relationship regarding cybervictimization. In other words, students with lower EI were more likely to suffer from cybervictimization and could experience negative repercussions on school success. Through EI training and addressing disruptive behaviors by focusing on school climate, classroom management, and discipline, we can create emotional regulation guidelines among students to eradicate disruptive behaviors.

## 1. Introduction

Information and communications technologies (ICTs) are now increasingly present in homes as they are highly accessible, both physically and economically. ICTs are a new tool for the expansion and fortification of interpersonal relationships; however, they can also be used to carry out actions of harassment and exclusion in relationships where power is abused [1].

The phenomenon of the necessary coexistence with other students in a school environment can camouflage face-to-face bullying behaviors and/or cyberbullying behaviors. Bullying is a deliberate behavior in which there is dominance and abuse based on an imbalance of physical, psychological, and social power [2].

### 1.1. Cybervictimization

The phenomenon of bullying has been the focus of research for more than four decades now [3]. The need for an amicable coexistence in the school space has given way to violence being transferred to digital spaces, finding in the technological world a strong ally for free circulation among the audience, perpetrators’ anonymity, diversification, and permanence of bullying incidents [4]. Bullying is a universal phenomenon [5], which some authors call “social cruelty online” [6], “social terrorism through new technologies,” or “epidemic,” according to some social networks. Currently, cyberbullying is mainly carried out using mobile telephony and the internet [7,8,9]. The arrival of smartphones has made cyberbullying easy for perpetrators as it integrates, in a single device, all the services provided by the network, including social networks (Facebook, Twitter, Instagram, Tumblr, YouTube). Smith et al. [10] categorize cyberbullying based on the technological methods of spreading harassment: Bullying by text messages (SMS) on mobile phones; bullying by making use of images/videos created using mobile cameras, for blackmail, dissemination, and multimedia messages; bullying perpetrated via emails (insulting or threatening emails); direct bullying with harassing calls via a mobile device; harassment in chat messages; intimidation through instant messaging (the most used applications in Spain are WhatsApp and Facebook); and intimidation through web pages with the purpose of defaming and ridiculing the victim.

Lapidot and Dolev [11] indicate that the roles of adolescents in traditional violence are the same as those in cyberbullying; however, in cyberbullying, the roles can be more complex than in most face-to-face confrontations, due to its peculiarities (anonymity, distance, rapid communication, easy accessibility to a wide-ranging audience) [12]. Comparing bullying and cyberbullying, there is controversy about whether cybervictims suffer more symptoms of depression and anxiety than victims of traditional bullying [13,14,15].

The perpetrators victimize the students with racist, sexist, and power dominance behaviors outside the school environment. The victims feel they are the object of aggression due to the envy they arouse among the aggressors due to the good grades they achieve or for having positive relationships with the teachers [16]. Bullies may feel threatened in a highly competitive society, perceiving the success of their peers as a threat. These aspects affect school integration: Difficulty in concentrating, interfering with school performance [17,18], refusal, and school absenteeism [19].

Victims of bullying are found to internalize their problems, they tend to have poor social skills, a tendency to isolate themselves [18], have low self-esteem, frustration, suicidal thoughts [17,20,21,22], and report greater defenselessness [23]. According to different studies, cyberaggressors display a lack of empathy, and a dependence on technologies and truancy [24]; in a high percentage of the cases, the perpetrator was known by the victim [25]; and individuals with disabilities were particularly at risk for cyberbullying [26].

Social support from friends acts as an important protector against cyberbullying [27]. Brighi et al. [17] analyzed the relationship between loneliness and cyberbullying/bullying using a sample of 5863 adolescents between the ages of 12 and 16. They concluded that victims of cyberbullying had a greater tendency to feel lonely. Loneliness is also associated with increased use of the internet and the problematic use of smartphones [28,29]. Adolescents who are victims of bullying try to find virtual friends in order to obtain emotional support to alleviate negative moods associated with loneliness, which can likely lead to greater risks of cyber abuse [30].

### 1.2. Emotional Intelligence (EI), Education, and Academic Performance

Emotional intelligence (EI), from the theoretical model of Salovey and Mayer [31], is defined as a set of specific skills that allow understanding, regulating, and experiencing emotions in a more adaptive way. It integrates verbal and nonverbal language, as well as the expression and regularization of the emotions of oneself and of others, that is, emotional awareness, which is essential for solving problems.

From this point of view, emotional education is fundamental and necessary for everyone in their day-to-day lives, because it helps to deal with personal and environmental changes. The development of our socio-emotional system allows us to pay attention and make decisions on the relevant aspects that direct our behavior toward achievements and objectives. Socio-emotional skills are necessary among students, both inside and outside the classroom [32,33]. Multidimensional analyses of EI in cases of cybervictimization have discovered that cybervictims pay considerable attention to their own emotions (emotional attention) but have difficulties understanding and managing them [34,35,36].

Additionally, some studies showed that EI had a unique power to predict the academic performance of students and the quality of their social interactions with peers [37]. Previous studies with cybervictims have suggested that online victimization may damage academic performance and self-concept [38]. Despite the importance of emotional skills, the role they have in school has been insignificant [39], bearing little influence on the training curriculum. Managing emotions in the school environment would represent an important dynamic component in education, which would help improve interpersonal relationships and academic performance [40]. MacCann et al. published a recent meta-analysis that explores how EI predicts academic performance; the analysis suggests that EI is an important predictor after intelligence and conscientiousness [40]. Emotional and social learning should be given the same importance as any other area of knowledge; the educational system should encourage this learning for the benefit of children and adolescents [41,42]. Some authors have proposed three mechanisms underlying the EI and academic performance link: Academic emotions regulation, building social relationships at school, and academic content overlap with EI [40].

The teaching and learning process is of great importance for the overall development of students. Therefore, the comprehensive training of students should include the development of both cognitive and social-emotional abilities. Learning to coexist peacefully includes affective, emotional, moral, and ethical components, so the construction of coexistence should not be based on punitive mechanisms to alleviate conflicts that ensure compliance with the rules established by the school [43]. Emotional education is important as it is a prevention mechanism against coexistence problems and cyberbullying problems [44]. It is necessary to create a positive climate in classrooms to stop interpersonal and relational conflicts, which in turn, will be conducive for emotional education in the classroom; students can thus learn to internalize the negative emotions they feel, analyze why they feel them, and learn alternative skills to violence [45]. Sometimes, due to little or no emotional reading of students and their behaviors by teachers, there is consolidation and permanence of these disruptive behaviors. In other words, EI would help create emotional regulation guidelines to eradicate or transform these negative behaviors [46].

The importance of EI in the educational field has been highlighted by various scientific studies. Based on research, it is evident that high levels of EI are related to greater self-esteem and leadership [47]; better prosocial behaviors [32,48]; social interaction and emotional regulation [49]; fewer disruptive behaviors and less school absenteeism [42]; better adaptation in the classroom, greater interpersonal sensitivity and empathy [32]; lower anxiety, rumination, and depression levels; higher levels of coping with problems; lower levels of suicidal ideation and suicidal attempts [22,32]; lower impulsive actions and aggressive temperament [50]; and better socio-academic adjustment in relation to academic performance [51].

In recent decades, bullying has become a major social problem at schools. In this day and age, when students consume more content online and have the majority of their social and academic interactions on these online media, regulating online activity is of utmost importance. There has been a noticeable increase in the factors affecting cyberbullying susceptibility during the COVID-19 pandemic as a result of an increase in social media and online gaming activity [25]. There is a set of school, social, cultural, and personal variables that can prevent or develop bullying behavior. It is important to analyze the social skills that may act as indicators, emphasize the importance of assessing cyberbullying systematically in all schools, and identify relevant variables or personal factors that programs should include to prevent and intervene in cyberbullying [52,53]. The goal of the present study is to provide new insights into the interactive role that EI and academic performance play in association with cybervictimization.

Taking into consideration the positive effects of EI, this study aimed to analyze: (1) The relationship between the EI of secondary school students and how it influences cybervictimization and vice versa; and (2) the repercussion of these variables on academic performance. According to the literature reviewed, it is hypothesized that a low EI has a negative impact on students’ cybervictimization and results in lower academic performance.

## 2. Method

### 2.1. Participants

A total of 3650 secondary school students participated in this study. The sampling method was no probabilistically incidental, depending on those centers to which access was obtained. The percentage of public schools were 76.2%, while 23.8% were private schools. The final sample in this study consisted of 3451 students, 1821 were boys and 1630 were girls. The age range was between 12 and 19 years (M = 15.73; SD = 1.30), and they all belonged to various secondary schools in the province of Almería (Spain).

### 2.2. Instruments and Variables

Cybervictimization: A risk-factor questionnaire was used to study the impact of cybervictimization [54]. This questionnaire was initially designed by Nocentini et al. (2010) [55]. The scale consists of 26 items that are divided into four factors: Impersonation, online exclusion, visual cybervictimization, and written and verbal cybervictimization. The instrument is scored on a 7-point Likert scale ranging from 1 (totally disagree) to 7 (totally agree). The study showed a Cronbach’s alpha value of 0.82. To test the factorial validity and criterion validity, and the internal consistency of the questionnaire, a sample of 2490 Spanish students from Secondary Compulsory Education was utilized [56]. This study provided a tool to assess cyberbullying with psychometric guarantees of reliability and validity in a broad, representative sample. This test is easy to administer, score, and interpret, and it has been widely used [24,53,56].

Emotional intelligence. The study used the adapted version of the Spanish Trait Meta-Mood Scale, TMMS-24 [57]. It assessed the participants’ ability to self-assess their own emotional intelligence, known as “Perceived Emotional Intelligence” [58]. The scale includes 24 items divided into three subscales of eight items each (the three subscales are perception or attention to feelings, understanding or emotional clarity, and regulation or repair of emotions). The items are rated on a 5-point Likert scale, where 1 = not at all and 5 = totally agree. In this study, Cronbach’s alpha value was 0.77.

Academic performance. Information related to the average global grades of the students was collected at the end of the course. The ratings ranged from 0 to 10, with 10 being the highest score. The teacher sent the information based on the data from the Seneca application.

### 2.3. Procedure

We contacted the educational centers and the parents’ associations to request authorization for the study during the second trimester of the 2017/2018 academic year. The sample of schools was selected from a total of 13 educational centers according to their availability to participate in the study. We explained the objectives and purpose of the study and ensured that we addressed all doubts and answered all questions. The students were all minors and, hence, were required to submit the informed consent form signed by their parents and/or legal guardians in order to participate in the study. The study allowed the participants to answer questionnaires anonymously by coding each student and their questionnaires with a number, and then delivering them in a sealed envelope. The data were collected during an allocated period of half an hour, before the beginning of classes, respecting the ethical standards established by the American Psychological Association (Reference: UALBIO 2017/010) and Helsinki Protocol. Students could refuse to answer if they found it difficult to do so. Eventually, 199 surveys were not coded in the database: When a student refused to answer all the items on the Cybervictimization Scale; or surveys that were suspicious in terms of the response patterns.

This study was part of a larger project in which the relationship between bullying or cyberbullying and the role of the different elements of emotional processing—perception, understanding, and/or regulation—as possible predictors of victimization in educational centers were being assessed [45].

### 2.4. Data Analysis

In this study, we conducted descriptive statistical analyses (mean and standard deviation), Pearson’s correlations, and reliability analysis using the SPSS v.24 statistical program (IBM, Armonk, NY, USA). Structural equation modeling (SEM) was used with AMOS v.19 [59] in order to analyze the predictive relationships of the factors.

**Figure 1:**
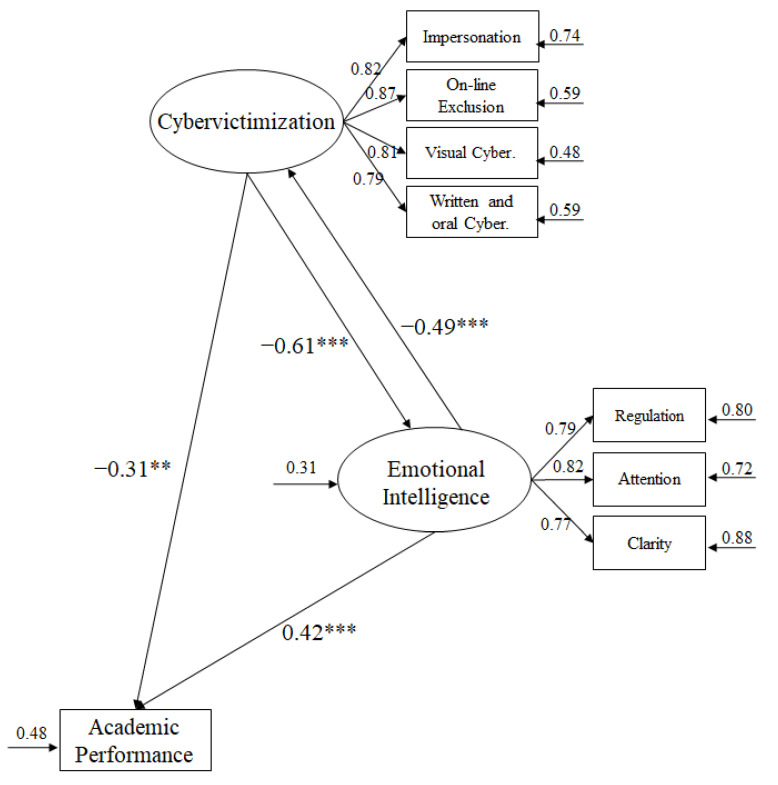
Structural equation model. The explained variances are shown on the small arrows. All parameters are standardized and statistically significant. Note: *** *p* < 0.001; ** *p* < 0.01.

To analyze the hypothesized model (Figure 1), the maximum likelihood estimation method was used together with the bootstrapping procedure. The estimators were considered robust as they were not affected by the lack of normality [60]. In order to accept or reject the tested models and CFA, various fit indices were used [49]: The chi-square coefficient divided by degrees of freedom (χ^2^/df), CFI (comparative fit index), IFI (incremental fit index), TLI (Tucker–Lewis index), RMSEA (root mean square error of approximation) plus 90% confidence interval (CI), and SRMR (standardized root mean square residual). Given that the χ^2^ is very sensitive to the sample size [61], we used χ^2^/df, considering values below 5 as acceptable [62]. Incremental indices (CFI, TLI, and IFI) show a good fit with values of 0.90 or higher [63], while the error rates (RMSEA and SRMR) equal to or less than 0.06 are considered acceptable [64].

## 3. Results

### 3.1. Preliminary Analysis

Table 1 shows the mean, standard deviation, reliability analysis through Cronbach’s alpha, and bivariate correlations. Correlation analysis shows a negative correlation between EI and cybervictimization and a positive correlation between EI and academic performance. That is to say, students with higher EI are less likely to suffer from cybervictimization and have better grades or are more successful at school. Reliability analysis using Cronbach’s alpha for each of the factors obtained scores above 0.70. These results are consistent with our predictions.

### 3.2. Structural Equation Model Analysis

Before testing the hypothesized model using SEM and analyzing the relationships between the variables of the model, a reduction in the number of latent variables was carried out, taking into consideration the complexity of the model [65].

More specifically, the latent variables used were cybervictimization including four indicators, namely, impersonation, online exclusion, visual cybervictimization, and written and verbal cybervictimization [54]. Finally, EI included three indicators, namely, attention to feelings, emotional clarity, and regulation of emotions [57].

The adjustment rates were adequate and were the following: *χ^2^* (18, *n* = 3451) = 53.67, *χ^2^/gl =* 2.98, *p* < 0.001, IFI = 0.97, CFI = 0.97, TLI = 0.97, RMSEA = 0.048. (IC 90% = 0.041–0.053), SRMR = 0.0.41. The contribution of each of the factors to the prediction of other variables was examined through standardized regression weights.

Next, the relationships between different factors that integrated the model are described (Figure 1):

(a) The cybervictimization negatively predicted EI (β = –0.61, *p* < 0.001) and academic performance (β = –0.31, *p* < 0.01). In other words, cybervictimization has a negative influence on the EI levels of students, affecting their academic performance negatively.
(b) Emotional intelligence positively predicted academic performance (β = 0.42, *p* < 0.001). Furthermore, EI negatively predicted cybervictimization (β = –0.49, *p* < 0.001). In other words, EI favors academic performance of high school students and makes them less susceptible to cybervictimization.

## 4. Discussion

The results of this study show there is a negative association between high EI rates and the possibility of being cybervictimized and school failure. Previously, it had also been observed that low EI rates were positively associated with cybervictimization [53]. The increase in values on some scales, such as adaptability, stress management, and interpersonal emotions, can involve the increased likelihood of various perceived manifestations of school violence [44].

Several studies have indicated that there is a significant relationship between perceived emotional intelligence (PEI) and school performance in secondary school [66,67,68]. Antonio-Aguirre et al. [69] indicate that the only dimension of the PEI that seems to affect academic performance is emotional attention—the negative effect is limited, and its influence is also limited to the female gender. In other words, paying too much attention to emotions would contribute toward enhancing ruminative thinking, placing personal and school adjustment at risk [70]. Although it establishes that EI and academic performance are correlated, Broc [71] describes that its degree of significance is so small that it could be related to factors such as psychological, family, and educational variables.

Authors such as Poulou [72] indicate that students who have high EI use, understand, and manage their emotions better; they display a lower degree of indiscipline and aggression, are less hostile in class, engage in prosocial behaviors, and achieve better academic performance. Students who have a greater ability to recognize the emotional state of others show better social relationships with peers, greater confidence, and better-perceived competence [45,73]. On the contrary, students with low emotional control and management are associated with high levels of victimization [74].

Other studies in the scientific literature highlight that positive relationships with teachers are associated with students’ perceived emotional support, which functions as a strong protector of disruptive behaviors [75], as well as a key element to prevent aggressive behaviors [76].

In short, a lack of EI skills affects the behaviors of adolescents [46]—it is positively associated with cybervictimization [53], low levels of well-being, and low psychological adjustment among young individuals [45].

Tokunaga (2010) [77], in addition to stating that cybervictims show the lowest academic performance, also indicates that they have high rates of school absenteeism. Likewise, Buelga et al. [78] report that cybervictims have less social support and less affiliation with their peers, and therefore experience greater loneliness, less academic self-esteem, and less involvement in school tasks; all these factors lead to worse academic achievement. Dropping out of school is significantly more common among children who have been cybervictimized [79].

Similarly, Beran and Li [80] and Sanz and Molano [81] consider that bullying and cyberbullying can have repercussions on school learning, which is reflected in poor concentration, worse school engagement, low grades, and increased absenteeism. However, they also explain that the fear of going to school can be the result of feelings of frustration, sadness, and fear caused by the aggression of perpetrators on the one hand, and the difficulty to face schoolwork, which can make them the object of teasing, ridicule, and intimidation, on the other.

The fear of aggression is the culprit of the problems associated with performance and school dropout, in addition to problems related to an emotional nature, self-confidence, mistrust, and so forth [82,83]. Díaz-Aguado et al. [16] suggest that establishing a good relationship with teachers or provoking envy among the peer group for good grades may be reasons why students are attacked, as the competitiveness established in school is related to success and can be perceived as a threat to those who do not have it.

Cyberbullying negatively correlates to schoolwork, and therefore, a favorable school climate is an important protective factor against online aggression [77,82,84]. Having an unhealthy relationship with school facilitates participation in aggressive behaviors such as cyberbullying [83], which in turn, generates greater anxiety about going to school, resulting in absentee behaviors [84].

This study has some limitations. First, based on self-reported data, the method of using questionnaires is simple and an inexpensive measurement instrument applicable to a large group of subjects; however, despite the fact that anonymity is a hallmark that guarantees the confidentiality of those surveyed, certain biases in the responses may occur as a consequence of social desirability. Second, although the sample is representative, it focuses on a specific training stage, and in turn, on a specific region of the Andalusian community, excluding other educational stages of equal scientific and social interest. Therefore, there is a need to carry out the generalization of the results with some caution. In future studies, it is advisable to have a replication model with other population samples from different geographical areas to verify the external validity of said results and include other compulsory educational stages.

On the basis of the practical implications of this work, and by relying on the situations described earlier, experts reveal that schools have an important responsibility in addressing the prevention of bullying and cyberbullying [85,86]; however, some are taking time to respond properly to this situation [87]. First, teachers underestimate the prevalence rates of cyberbullying because it is less visible to them [88] and because most of the cases originate outside the school and are considered outside the framework of their responsibility [89,90]. It is necessary to promote programs that support the socio-emotional development of adolescents and focus on encouraging and empowering children and young people [91]. Therefore, training or education in EI issues should be integrated into bullying and cyberbullying education in schools [44]. It is important to detect these incidents early to propose effective measures for prevention and treatment. Such empowerment would help cybervictims and would prevent the spread of the harm resulting from the viral nature of this type of bullying [12]. These programs should place emphasis on observers, raising awareness of the importance of their role in eradicating cyberbullying in all its forms. Promoting emotional education in the classroom will make students understand their emotions and those of their classmates better, providing them with alternative skills to manage conflict, aggression (emotional regulation), or anger, which are the origins of many violent behaviors [45]. Applied in educational settings, these programs can help to decrease cybervictimization and cyberaggression, while promoting socioemotional development by stimulating social adjustment, self-esteem, prosociability, comprehension, and expression of emotions, empathy, agreeableness, and so on [24].

## 5. Conclusions

A low EI value predicts greater likelihood of suffering from cybervictimization, as well as lower academic performance. Through training in EI and addressing disruptive behaviors by focusing on school climate, classroom management, and discipline, we can create emotional regulation guidelines among students to eradicate disruptive behaviors. These programs will likely reduce the probability of cyberbullies, facilitating more prosocial behaviors and better psychological adjustment of young people, which will positively affect their grades.

## Figures and Tables

**Table 1:** Descriptive statistics, reliability analysis, and correlations between all variables.

Factors	M	SD	*α*	1	2	3
1. Cybervictimization	1.15	0.24	0.82	-	−0.45 ***	−0.28 ***
2. Emotional Intelligence	3.24	0.66	0.77		-	0.42 ***
3. Academic Performance	6.07	1.75	-			-

*** *p* < 0.001.
